# A pilot qualitative study to explore stakeholder opinions regarding prescribing quality indicators

**DOI:** 10.1186/1472-6963-12-191

**Published:** 2012-07-07

**Authors:** Liana Martirosyan, Joekie Markhorst, Petra Denig, Flora M Haaijer-Ruskamp, Jozé Braspenning

**Affiliations:** 1 Department of Clinical Pharmacology, University Medical Centre Groningen, University of Groningen, Groningen, the Netherlands; 2 Centre for Quality of Care Research (WOK), University Medical Centre St Radboud, Nijmegen, the Netherlands

## Abstract

**Background:**

Information on prescribing quality of diabetes care is required by health care providers, insurance companies, policy makers, and the public. Knowledge regarding the opinions and preferences of all involved parties regarding prescribing quality information is important for effective use of prescribing quality indicators.

**Methods:**

Between June and December 2009 we conducted semi structured interviews with 16 key-informants representing eight different organizations in the Netherlands involved in healthcare quality measurement and improvement. The interview guide included topics on participants’ opinions and preferences regarding existing types of prescribing quality indicators in relation to their aim of using quality information. Content analysis methods were used to process the resulting transcripts following the framework of predetermined themes.

**Results:**

Findings from this qualitative study of stakeholder preferences showed that indicators focusing on undertreatment are found important by all participants. Furthermore, health care providers and policy makers valued prescribing safety indicators, insurance companies prioritized indicators focusing on prescribing costs, and patients’ organization representatives valued indicators focusing on interpersonal side of prescribing. Representatives of all stakeholders preferred positive formulation of the indicators to motivate health care providers to participate in health improvement programs. A composite score was found to be most useful by all participants as a starting point of prescribing quality assessment. Lack of information on reasons for deviating from guidelines recommendations appeared to be the most important barrier for using prescribing quality indicators. According to the health care providers, there are many legitimate reasons for not prescribing the recommended treatment and these reasons are not always taken into account by external evaluators. The latter may cause mistrust of health care providers towards external stakeholders and limit the use of PQI in external quality improvement programs.

**Conclusion:**

Prescribing quality indicators are considered to be an important tool for assessing quality of provided diabetes care by all participants, although the preferences for specific types of indicators may differ by stakeholder depending on their user aim. Introduction of information systems to register the reasons for deviating from the recommended drug treatment may contribute to a more widespread use of PQI for assessment of provided health care quality to diabetic patents. This study identified the potential preferences regarding quality indicators for diabetes care, and this could be used for development of questionnaires to conduct a survey among a larger group of participants.

## Background

Insight into the quality care is demanded by healthcare providers, payers, and patients. These different stakeholders use quality information for different purposes such as internal quality improvement, cost containment, and accountability. There is general agreement that due to varying aims of using quality information, the different stakeholders have specific preferences for the type of quality information [1-3]. However, not much is known about their actual preferences. In this study, we explored the opinions and preferences of different stakeholders regarding prescribing quality indicators in diabetes care.

Appropriate drug prescribing has been recognized as an important quality of care issue in the management of chronic conditions, such as type 2 diabetes mellitus (T2DM). Diabetes is a chronic disease with a dramatically increasing prevalence throughout the world [4]. Appropriate pharmacological treatment of diabetes and related risk factors helps to reduce complications in patients with T2DM [5].

To measure quality of prescribing in T2DM, a large number of prescribing quality indicators (PQI) has been developed [6]. Among the existing PQI there are distinct types of PQI that address different aspects of prescribing, *i.e.* PQI focusing on undertreatment, safety, first choice medication, and costs. The PQI focusing on undertreatment look into prescription of medication in patients with a specific diagnosis or elevated values of clinical measurements. The safety PQI address prescription of medications in those with contraindications or side effects. The first choice PQI assess prescription of a medication within its drug class based on the guideline recommendations, and PQI focusing on costs assess prescription of the cheapest medication within its drug class. Despite the increasing number, the PQI for T2DM management are largely underrepresented in national sets of quality measures that are used for external accountability in different countries. For example, the National Voluntary Consensus Standards for Adult Diabetes Care includes two PQI focusing on management of diabetic patients [7], and only these PQI were included in the Health Employer Data Information Set (HEDIS) of measures [8]. The Diabetes Quality Improvement Project, which was implemented in the United States as a comprehensive set of national diabetes quality measures, did not include any PQI for internal quality improvement or for accountability [9]. The PQI are also underrepresented in the Quality and Outcome Framework set of quality indicators in the United Kingdom with only one PQI relevant for diabetes care [10]. The Australian national set of diabetes indicators does not include any explicit PQI [11]. On the other hand, in some countries, for example, the United Kingdom and Australia, there are sets of internal quality indicators exclusively focusing on prescribing issues [12,13]. In the Netherlands, a similar situation exists with PQI mainly being used for internal quality improvement and only a few used for accountability purposes [14,15]. The reasons why PQI are not widely used by different stakeholders, especially for external quality assessment, are not obvious. It is not clear if stakeholders such as health insurance companies or policy makers do not find the PQI useful for their work or if there are obstacles in implementation of quality improvement programs with the use of PQI.

Previous studies investigating the preferences for PQI focused mostly on needs of healthcare providers (HCP). It was found that PQI based on detailed patient clinical information are preferred to those based on aggregated data [16], and that physicians rank evidence-based PQI higher than those based on costs [17]. However, there is scarce knowledge regarding the types of PQI that are found relevant by other stakeholders. Furthermore, little is known about the preferred format of presenting the scores of PQI. For instance, it is possible to focus either on numbers of patients receiving appropriate care or on patients receiving inappropriate care. Also, indicators can represent one specific item of care or can average several items into a composite score. The use of PQI could be more effective if we had a better understanding of ways to present quality information that are most meaningful to the involved stakeholders.

The aim of the current pilot study was to facilitate the future selection of prescribing quality indicators for management of type 2 diabetes by conducting interviews with the key representatives of several relevant organizations involved in development of such measures in the Netherlands. We explored whether and why the PQI are considered a relevant part of quality assessment of T2DM care, and which types of PQI should be included in quality improvement programs for diabetes according to different key representatives. In addition, we wanted to elicit the preferred way of receiving quality information as well as the perceived barriers regarding PQI use.

## Methods

### Study population

The present study draws on 16 semi-structured interviews with professionals involved in quality of care assessment using quality indicators. These participants worked for eight organizations responsible for healthcare policy development and involved in quality measurement or improvement in the Netherlands. The participating organizations represented (1) patient representatives, (2) healthcare providers, (3) payers, and (4) the Ministry of health, welfare and sport of the Netherlands (Table 1).

**Table 1:** Participating stakeholders and organizations

**Stakeholder**	**Organization**	**Positions of interviewed persons**
Patient organization	The Federation of Patients and Consumers’ Organizations	Senior policy officer
		Medical advisor
Health care providers	Dutch Institute of Health Care Quality Improvement	Senior advisor/diabetologist
	Dutch College of General Practitioners	Authors of national diabetes guidelines for primary care/primary care physicians
	Dutch Diabetes Federation	Diabetologist
		Diabetes nurse
	Royal Association for the Advancement of Pharmacy	Senior researcher/pharmacist
	Scientific Institute of Dutch Pharmacists	Senior manager/pharmacist
	Community health care providers	Primary care physician
		Diabetes nurse
Payers	Health insurance companies*	Health program manager
		Health care purchaser
		Medical advisor
Policy makers	Ministry of health, welfare and sport/Dutch	Senior inspector
	Health Care Inspectorate	Primary health care inspector

*We have included three different insurance companies covering different geographical regions in the country.

We have chosen to conduct in-depth interviews, as not much is known about the reasons for choosing and using different types of PQI by relevant stakeholders, *e.g.* insurance companies or health care professionals. Considering the lack of information on this issue made it impossible to define specific categories of potential answers and to conduct a survey. We were interested in specific reasons for using distinct types PQI, *i.e.* PQI focusing on undetreatment, safety, first choice drug and costs, by different stakeholders, barriers for implementation of PQI in the daily practice and the future they see for the PQI for their organization. In-depth interviews were therefore considered relevant to answer the research questions in this study.

Purposive sampling was used to identify the participants from each organization, *i.e.* senior staff members who were engaged in quality of care measurement or improvement using quality indicators within the organization. We have contacted our respondents directly or identified them through other employees of the included organizations by asking them to suggest the senior staff members whose tasks are most closely related to quality assessment or improvement with use of quality indicators. For example, within the Ministry of health, welfare and sport we have contacted the health inspectorate, an organization within the ministry responsible for the quality and safety of provided health care in the Netherlands. For patient representatives, we have selected the Federation of patients and consumers’ organizations, as this organization is dealing with patient rights and preferences in the Netherlands in different disease areas including diabetes. We did not aim to include patients with diagnosis of T2DM the questions included in the semi-structured guide required professional knowledge regarding different types of prescribing quality indicators and their use for quality assessment. For the relevant sample of the health care providers, we have selected professionals (diabetes nurses, primary care physicians, specialists, and pharmacists) who were the active members of several professional organizations involved in health care quality improvement in the Netherlands (Table 1). Another stakeholder, payers, was represented by three different health insurance companies that are operating in different geographical regions of the country. All participants received a letter containing information about the aim and methodology of the study.

Under Dutch law, ethical approval is not required for studies using face-to-face interviews. Therefore, this study was exempted from approval of the medical ethics committee.

### Interview guide

A semi-structured guide was used that included open-ended questions about aims of collecting and using quality information, and more specific questions related to opinions about and preferences for different types of existing PQI, the preferred way of receiving quality information, and factors limiting their use. The following topics were covered in the interview guide: a) Current usage and aims of using prescribing quality indicators; b) Opinions regarding the relevance of including PQI on assessment of quality of diabetes care; c) Opinions regarding and prioritization of existing types of PQI, *i.e.* focusing on undertreatment, safety, first drug choice, and costs; d) Opinions regarding formulation of the PQI (positive versus negative score); e) Opinions regarding aggregation level of the PQI; and f) Perceived barriers for implementation of PQI. After eliciting opinions regarding existing types of PQI, on undertreatment, first choice-drug, safety and costs, we have asked the participants to choose the type(s) of PQI that were most relevant for their work and the reasons for their prioritization. The instrument was pilot-tested prior to the data collection.

### Data collection

Data collection was carried out between July-December 2009. The interviews were conducted face-to-face for 13 participants and by telephone for three participants who preferred to be interviewed this way. Interviews lasted on average 1.5 hours, ranging from one to two hours. The face-to-face interviews were conducted by two researchers; one was conducting the interviews and another one was making notes. The interviewer asked open-ended questions to reveal participants’ views and preferences, and then probed for clarification or to explore new themes as they appeared. All interviews were recorded on digital recorders with permission of participants. All participants gave a written consent to participate in the interview. To ensure the accuracy of our data we used several techniques. First, the interviews were translated verbatim independently by the two researchers present at the interviews. The transcripts were compared and disagreement was resolved through discussion. Next, the accuracy of all transcripts was checked against the original recordings by an independent researcher. Finally, the transcripts of interviews were sent back to the interviewees who were asked to check their consistency and accuracy before the analysis.

### Analysis

The analysis of data began in parallel to data collection. The semi-structured interview guide was only slightly adjusted in the course of interviews, *i.e.* the sequence of some of the questions has been adjusted to improve the logical flow of the information, and we had the same topics and questions covered in all interviews.

The transcripts of each of the interviews were analyzed using a content analysis [18] according to a predetermined framework. This framework was based on the known knowledge regarding the use of PQI by different stakeholders as described earlier in this paper. In this framework, opinions of different stakeholders regarding the use of PQI were divided into the following four main themes: (1) relevance of PQI for measuring the quality of provided diabetes care; (2) opinions and preferences for specific types of PQI; (3) potential barriers for using PQI; (4) preferred ways of receiving prescribing quality information. Within these pre-defined themes, we identified parts of text that related to the same concepts. Next, we coded data by giving descriptive code-names to these concepts. Later, we grouped similar codes under the larger, main categories. Finally, we re-organized our data by stakeholder in order to identify differences and similarities per stakeholder.

The transcripts of each interview were initially coded by the first co-author with regular discussion of an emerging framework for data coding with other co-authors followed by the second co-author’s review of coding. We used qualitative analysis software (ATLAS.ti Win 6.1) to facilitate organization of data into codes, categories and themes [19].

## Results

### Usage and aims of prescribing quality indicators by the stakeholders

All participants with the exception of the representatives of the patient organization used some sort of PQI for T2DM management. Primary care physicians and diabetologists use PQI for T2DM management for internal quality improvement initiatives such as peer review. The representatives from the Dutch Health Inspectorate are primarily interested in investigating and following up on problems encountered in medical institutions. Therefore, they mainly use outcome quality indicators to identify the healthcare institutions that do not meet the minimal levels of predefined standards of quality. Recently, however, they launched a set of PQI for pharmacies to improve pharmaceutical care. Pharmacists in the Netherlands are increasingly being involved in pharmaceutical care and prescribing quality assessment, and are encouraged to search for patients not receiving the optimal treatment and alert physicians. For these purposes, they use various PQI for T2DM management and report the scores of these indicators to the Inspectorate. In addition, pharmacists report on PQI focusing on costs of medication to health insurance companies. Health insurance companies primarily collect PQI focusing on costs to provide financial incentives to the HCP that keep prescribing costs low. Finally, the patient organizations collect quality information to support patients when making HCP choices, and to develop policies where patients' preferences are taken into account. Currently, no PQI are used by patient organizations.

### Relevance of using PQI for assessing the quality of provided diabetes care

#### PQI are an integral part of diabetes quality indicators set

Representatives of all included stakeholders stressed the importance of combining PQI with other quality indicators of diabetes care to obtain a comprehensive picture of provided quality. It was noted that since diabetes is a chronic disease, there is a large number of factors that determine the final outcome of the treatment, and it is precisely the combination of all relevant processes that defines quality of diabetes care.

"“The most important is overall treatment of an individual patient; therefore prescribing should be always evaluated in combination with other processes and outcomes of care.” Diabetes nurse"

"“The prescribing quality for diabetes is as important as the eye or foot exam. All aspects of treatment should be taken into consideration.” Medical advisor/health insurance company"

Some participants strongly argued against the use of PQI alone without other quality indicators, as different quality indicators of diabetes care are highly interrelated, *i.e.* the physicians need to measure and register certain clinical values in the records, so later they are able to make decisions about the treatment choices.

"“I think that PQI should be seen as an integral part of quality of diabetes care and should never be considered separately from other quality indicators.” Senior researcher/Royal Association for the Advancement of Pharmacy"

"“…The PQI are only important if combined with other quality indicators. Total care is more important than the prescribing patterns alone.” General practitioner"

### PQI reflect actions of healthcare providers

The HCPs noted that there is a lot of attention on quality indicators focusing on measurement and registration of HbA1c and other risk factors. Prescribing indicators, however, are more relevant as measures reflecting the actions of healthcare providers in response to observing elevated risk factors, as eventually most of diabetic patients require pharmacotherapy.

“Measurements of the blood pressure or cholesterol are process indicators. These process indicators are prerequisites for follow up actions.. Currently, a lot of attention is still being paid to this type of indicators [focusing on registration of measurements] that are actually not very important. It is more important to know what the health care providers do after observing elevated values of risk factors, and prescribing quality indicators play an important role here.” General practitioner/Dutch College of General Practitioners

### PQI reflect scientific evidence

All participants believed that it is important to include PQI in quality improvement programs, because PQI usually reflect the evidence-based recommendations from clinical guidelines. Moreover, the HCPs stated that prescribing is the most evidence-based part of diabetes treatment, as the other processes of care, *i.e.* registration of clinical measurements, lifestyle modification or diet are not as well researched in relation to patient outcomes as prescribing.

"“…the evidence of patient-targeted educational programs in relation to clinical outcomes is lacking, while prescribing is supported by harder evidence, and it is a very important part of diabetes treatment.” General practitioner/Dutch College of General Practitioners"

### Opinions regarding and prioritization of existing types of PQI

#### PQI focusing on undertreatment

PQI focusing on undertreatment were found essential by representatives of all stakeholders (Table 2). A room for improvement and reflection of guidelines were the most frequently mentioned reasons for being interested in these PQI. The HCP found these PQI very relevant for their work, because undertreatment of diabetic patients remains a major problem in a practice.

**Table 2:** Potential relevance of existing types of PQI for different stakeholders

**Stakeholder**	**Undertreatment**	**Safety**	**First choice drug**	**Cost**	**Communication**
Patient organization	✓				✓
Health care providers	✓	✓			
Payers	✓			✓	
Policy makers	✓	✓			

"“I think that this type of information [information on undertreatment] is really important, because clinical inertia [initiation or intensification of therapy when indicated] is a big problem in treatment of type 2 diabetic patients. A good example is statines that are hugely underprescribed in patients with T2DM.” Diabetolgist/Dutch Institute for Healthcare Improvement"

Participating pharmacists noted that they prioritize these PQI, because it is easy to improve the scores of PQI focusing on undertreatment due to a large number of undertreated patients.

"“It is quite easy to improve on this type of indicators, and of course it is always nice for pharmacists to dispense more medications.” Senior manager/Scientific Institute of Dutch Pharmacists"

Representatives from health insurance companies mentioned that they find these PQI very important because they reflect the timeliness of the start and intensification of treatment, and because undertreatment results in complications that add to the healthcare costs in long run.

"“When patients need certain treatment, they should be able to receive that treatment. In the end, poor care is more costly.” Healthcare program manager/health insurance company"

The representatives of the Health Inspectorate and the patient organization considered undertreatment of patients to be equal to the “wrong treatment”, and noted that patients who are in need of therapy have the right to be prescribed the recommended treatment.

"“We are very much interested in PQI focusing on undertreatment, as it [undertreatment] can harm patients on the long run.” Primary Healthcare Inspector/The Healthcare Inspectorate"

#### PQI focusing on safety

PQI focusing on safety were prioritized by the HCPs and the key informants from the Inspectorate. The HCPs mentioned that a large number of diabetic patients have kidney function impairment, and such patients are at higher risk of experiencing side effects from the prescribed medication. Therefore, safety issues in diabetic patients with kidney impairment are high on their agenda. Besides, the HCP noted that the average diabetic patient requires multiple drugs and has other conditions besides diabetes that put him more prone to experiencing side effects. The HCP found safety PQI focusing on drug-drug and drug-disease interactions to be very important for assessing quality of provided diabetes care.

"“Safety is first, because I do not want to harm patients with the medication. Using safety PQI that may prevent potential harm is very important for internal quality improvement.” General Practitioner/Dutch College of General Practitioners"

"“That is very important; as I see a lot of problems in patients with kidney disease, or patients with swollen ankles who get NSAIDs [non steroid anti-inflammatory drugs] in high doses, and the kidney function collapses because of that. I think interactions between different [drug] classes are very important.” Diabetologist/Dutch Institute of Healthcare Improvement"

Pharmacists prioritized the PQI focusing on safety, as they felt that they have the best knowledge on safety of medication and, therefore, they have the capacity to have a direct impact on improvement of patient’s safety in relation to prescribed medication.

The representatives from the Inspectorate prioritized PQI focusing on safety, because pharmacotherapy involves many errors and suboptimal decisions, and patients will directly benefit from the improvement in prescribing safety. In addition, it was noted that safety of provided healthcare is one of the most important criteria for the Healthcare Inspectorate.

The participants from health insurance companies felt that safety of prescribed medication should remain a professional area to be monitored and improved internally. Although they accepted the importance of safety PQI for diabetes care, in their opinion, judgments on these indicators require professional knowledge and skills they lack.

"“It is important that insurance companies do not take the place of healthcare providers and do not interfere too much in prescribing process. I believe that professionals are perfectly able to improve the safety of prescribing themselves.” Healthcare purchaser/Health insurance company"

#### PQI focusing on first choice drug

The value of PQI focusing on the first choice drug as seen by the key informants representing different stakeholders was that they usually reflect guideline recommendations and include a safety component. However, no interviewed key informant found these PQI very relevant for their own aims (Table 2). The health insurance representatives referred to these PQI as being only important in situations when the first choice drug recommendations implied prescription of cheaper medication. The representatives from the Inspectorate mentioned that although these PQI usually have good face validity, they do not always reflect prescribing quality. In particular, they noted that a high grade of evidence is not always available to guide an evidence-based drug choice, and in such situations the final drug choice needs to be made by physicians. The HCPs had a similar opinion about the PQI focusing on the first choice drug. One participant argued that these PQI might be used for internal purposes and never for external accountability. The HCP argued that the first choice drug recommendations available in clinical guidelines cannot be applicable to all patients, as there will be always some patients that experience side effects, have contraindications, or refuse the recommended medications, making these PQI very sensitive to patient case-mix.

Another limitation of these PQI mentioned by the representatives of the Inspectorate and the HCPs was the dynamic nature of the evidence supporting first choice drug recommendations. These participants noted that the recommendation in guidelines can change, because of emerging evidence suggesting another first choice drug.

"“Fifteen years ago, a professional was considered incompetent if he used it [metformin], as there were too many side effects, whereas now it has become a first-choice medication, which, of course, may change again.” Senior Inspector/The Healthcare Inspectorate"

In addition, pharmacists mentioned that information on first choice drugs is not so crucial, as it often refers to the choice from two drugs that can both be quite good, and therefore, the difference is not as big as between safe and unsafe therapy.

#### PQI focusing on cost

PQI focusing on costs related to prescribing were recognized as being relevant for the healthcare system by all participants but they were prioritized only by the representatives of health insurance companies. Key informants from other stakeholder organizations, while accepting the importance of reducing costs attributed to prescribing, felt that it is not their responsibility to control costs and mentioned that costs should not be the main factor in the prescribing process. The participants from the patient organization believed that costs do not have a relation to quality, since quality means meeting the needs of an individual patient without considering costs. Similarly, representatives of the Inspectorate noted that PQI focusing on costs are hardly interesting for them as these PQI do not reflect quality of provided care.

"“Costs and quality go together, but costs are not a priority for the Inspectorate. For instance, if we know that a certain medication is more effective than another, less expensive medication, we prefer the more effective medication in spite of the higher costs. Indicators relating to costs are hardly interesting.” Senior Inspector/The Healthcare Inspectorate"

In general, all HCPs including pharmacists mentioned that is not their priority to know if the cheapest medication has been prescribed. However, pharmacists mentioned that they do collect and report on PQI focusing on costs, as this is requested by health insurance companies. All stakeholders that did not prioritize PQI focusing on costs agreed that prescribing a cheaper medication is only relevant in a situation when several drugs with similar effectiveness are available.

#### PQI focusing on communication between HCP and patients

Representatives of the patient organization noted that although there will always be patients who would like to know the very detail about provided quality of care, for an average patient it is difficult to judge the quality of care with the existing PQI. In addition to the PQI focusing on undertreatment, they prioritized a different type of PQI that would reflect effective communication between HCPs and patients regarding the prescribed medication. The aspects considered as important were related to patients’ participation in the treatment process, self-management, patients’ empowerment and motivation to comply with the prescribed treatment, and provision of sufficient information about prescribed medication in an acceptable and understandable way.

"“It is important to measure whether the [treatment] decisions have been shared with patients, for instance when introducing insulin treatment… Doctors should motivate patients to comply with the therapy and to try achieving the improved health status”. Senior policy officer/Federation of Patients and Consumer Organisations in the Netherlands"

"“The HCP should provide information about the prescribed medication, for example common side-effects to be expected, *etc.* And most importantly, patients should believe in the medication prescribed by a doctor.” Medical advisor/Federation of Patients and Consumer Organisations in the Netherlands"

### Potential barriers for use of PQI

#### Reasons for deviating from the recommended treatment

The most frequently mentioned barrier for implementing PQI was the concern that reasons for not prescribing the recommended medication are ignored. The strongest opinions were expressed by the HCPs, who noted that many PQI are very sensitive to patient case-mix. Several patients may encounter side-effects to the recommended drugs, have contraindications, or simply refuse certain types of medication due to, for example, negative experiences in the past or influence of the media. There was a general concern raised by the HCPs that external bodies expect very high scores on indicators reflecting guideline recommendations, and that the external evaluators may not take into account all the legitimate reasons for not prescribing a recommended treatment to certain patients.

"“Those things [side effects, contraindications and patient refusals] are part of the equation and were never understood by the government, insurance or whatever. They say: “Oh, your score on prescription of metformin is only 80%. That is very bad, it should be 100%”… Ok, it is right it should be as high as possible, but there can be very good reasons not to prescribe the recommended treatment and that holds true for many other prescribing quality indicators”*.* Diabetologist/Dutch Institute for Healthcare Improvement"

The representatives of the Inspectorate and insurance companies, however, did recognize that there might be many legitimate reasons for deviating from the recommended treatment. They mentioned that usually they do not have insight to reasons for deviations, and knowing this information would be very helpful for fair prescribing quality assessment.

#### Prescribing is a professional area

Representatives of insurance companies and the Inspectorate mentioned that for some specific types of PQI, such as focusing on safety, one would need sufficient professional knowledge to be able to judge the scores of the indicators. It was mentioned that lack of such knowledge could be solved by setting up an expert panel. According to the representatives of the Inspectorate, the main reason why the information on PQI is not yet collected from medical practices is a traditional belief that prescribing is a professional domain in which the Health inspectorate should not interfere unless obvious problems are encountered. The majority of the HCPs supported this view, as they believed that they are capable of improving prescribing quality themselves by conducting internal audits and peer-review without external interference.

"“Measuring the quality of prescribing is new and tricky for the Inspectorate. Some health inspectors believe that prescribing is a professional domain in which they should not interfere as they do not have sufficient knowledge. It is, of course, possible to organize an expert panel, but still the most important point is whether or not prescribing should remain the responsibility of the professionals. “Senior Inspector/The Healthcare Inspectorate"

"“Doctors will not trust external evaluators for prescribing quality assessment. Cost control is more or less accepted, but quality assessment is not. We are capable of improving quality ourselves without interference of the Inspectorate or other external bodies.” Community primary care physician"

#### Operational feasibility

Although all participants agreed that currently available health records have the potential to provide information on the most important prescribing quality issues, operational feasibility was a frequently mentioned barrier to the actual use of PQI. In particular, many mentioned that the feasibility of calculating safety PQI is hampered, as this type of PQI require additional clinical information, such as kidney function, co-medication, *etc.* This type of information is not always available or easily retrievable from the registration systems. This was a particularly important issue for pharmacists. They believed to have the best knowledge related to the medication safety issues, but their involvement in prescribing quality improvement was limited by the lack of sufficient patient clinical data in the pharmacy registries.

“Prescription of certain medications in patients with impaired kidney and liver function requires a special attention, and pharmacists can be helpful in monitoring this. However, pharmacists normally do not have an access to such clinical information.” Senior researcher/Royal Association for the Advancement of Pharmacy

Another important piece of information that in particular the HCPs and representatives of the patient organization felt was lacking, was the documentation of patient preferences for treatment choices, and socio-economic factors that might influence patient preferences. The HCPs stressed the relevance of developing a registration system where this type of information could be entered in a systematic way, so it could assist shared-decision making and taking into account patient preferences.

Finally, the HCP mentioned that the numbers of PQI developed for different clinical areas is growing markedly, and there is a great time burden for them to deal with such a large number of PQI. The same problem was identified by the key informants from the Inspectorate, who mentioned that a large number of existing indicators related to quality of prescribing makes the choice of the most relevant PQI difficult.

### Preferred ways of receiving the prescribing quality information

#### Formulation of scores

A positive formulation of PQI scores was preferred by most participants and in particular by the HCPs. All HCPs mentioned that it is always better to start from the figures that focus on numbers of patients who are well treated, and only as a next step to discuss areas that need improvement. Starting with negative figures was thought as creating “a blaming culture” that can discourage and demotivate the HCPs from participation in quality improvement programs or from providing transparent data on quality.

"“Try to be positive … What would be the incentive for those who perform worse than the others? If you put good guys in front and bad gays in the back, you focus on the bad ones…Negative formulation creates chaos and negative attitude.” Diabetologist/Dutch Institute for Healthcare improvement"

Key informants from insurance companies were well aware of this fact, and preferred using positive figures to improve the chances of making successful contracts with HCPs.

“Positive formulation is important for creating a positive and encouraging atmosphere in the communication process with the health care professionals. If you go to the professionals and start the discussion with presenting figures that represent a good performance, this has a stimulating effect.” Healthcare program manager/Health insurance company

#### Aggregation level

When asked about preferences for aggregating PQI, all participants mentioned that both “composite” and individual scores are useful. The HCPs preferred a “fold out” system for internal quality assessment, where first a composite score, *i.e.* an average of all PQI is used to get a comprehensive overview, and next, it is folded out to the individual PQI level to identify potential areas for improvement. For external reporting, the HCPs preferred using only a composite score. The main underlying reason was the fear that external stakeholders may misinterpret the scores on individual PQI because they may fail to acknowledge the possible legitimate reasons for not prescribing the recommended treatment to certain patients. All external stakeholders, however, preferred to be informed on both aggregated and individual indicator level using a “fold out” system. The use of composite scores was considered to be convenient by providing a quick overview and eliminating the necessity of dealing with too many quality indicators. Despite this, the composite score was never considered informative enough. Information on an individual PQI level would be desired eventually, since only individual PQI scores ensure transparency of the provided care and identify areas that require special attention. Several participants mentioned that composite scores should ideally aggregate only indicators focusing on a similar topic, for example, safety or undertreatment.

## Discussion

Our study showed that all participants representing different stakeholders thought that PQI are reflecting an important part of the treatment process of T2DM patients and that PQI should be included in the T2DM quality improvement programs. All stakeholders found PQI focusing on undertreatment to be very relevant for their own user aims. In addition, the interviewed HCPs and the Inspectorate key informants prioritized PQI focusing on safety. No participants found PQI focusing on the first choice drug important for their aims. For the remainder, the participants representing different stakeholders had differing priorities for the types of PQI. Health insurance companies prioritized PQI focusing on costs, and the patient organization valued quality indicators that would reflect effective communication between patients and HCPs. Important barriers for using PQI were concerns that legitimate reasons for not prescribing the recommended treatment are overlooked, and relevant clinical information is not always available for adequate prescribing quality assessment. As for the preferred way of presenting scores of the PQI, we found that a positive formulation of indicators is very important for encouraging the HCPs to participate in prescribing quality improvement programs. A composite score averaging several PQI was considered a convenient way to start the process of prescribing quality assessment by all participants, but scores on individual PQI were always preferred to inform quality improvement initiatives.

### PQI are important tools for assessing quality of diabetes care

We found that all participants stressed the importance of including PQI for assessment of diabetes care. The reasons brought forward by different stakeholders’ key informants included the relatively high level of evidence available for PQI compared to other quality indicators, and prescribing being a vital component of T2DM management. Although carefully managing diet, exercising, and self-monitoring contributes to improved health outcomes in diabetic patients [20,21], for the majority of patients these interventions alone are not usually sufficient. To avoid or minimize chronic diabetic complications, some sort of pharmacological treatment will then be necessary because of progressing nature of the disease [22].

Furthermore, there is an increasing interest from the Healthcare Inspectorate in receiving information on quality and safety of medication use in the Netherlands. The recent endorsement of prescribing indicators to be used by pharmacies in the country, confirms this trend. Similarly, the National Quality Forum in the United States acknowledged that there are too few measures available to improve the quality and safety of medication use and management, and endorsed 18 prescribing quality measures as a starting point. These measures focus on managing over-the-counter and prescription medication related to several conditions including diabetes [23].

Our results indicate that PQI focusing on undertreatment can be included in a uniform set of quality indicators to be used by all stakeholders. For the rest, the stakeholders had differing preferences specific to their user aims, and should used customized sets of PQI to reflect their own interests and priority areas. We have found that PQI focusing on costs were not interesting for the HCPs, and this is consistent with findings from other studies [17,24,25]. In the past, PQI on costs have been a part of internal quality improvement programs [26-28]. Auditing such information on prescribing appears to be less relevant for HCP nowadays, probably because health insurance companies now use different (reimbursement) strategies to control the prescribing costs.

### Preferences of stakeholders regarding PQI

The PQI focusing on first choice drugs were not prioritized by any stakeholder representative. The first drug choice recommendations are an important component of many clinical guidelines. However, PQI reflecting these recommendations are more likely to be affected by patient case-mix and the changing evidence base. In comparison with the PQI that focus on undertreatment and refer to prescribing of any drug from a certain class, the PQI focusing on first choice drug look at prescribing of a specific drug within a class. Therefore, it is more likely that the scores of such PQI are lowered due to some patients experiencing side effects or having contraindications to this specific drug. In addition, the changing nature of evidence supporting the PQI focusing on first drug recommendations hampers comparisons of prescribing quality scores over time.

Representatives of the patient organization and the interviewed HCPs identified an existing gap regarding information on interpersonal side of prescribing quality, *i.e.* shared decision making and respect for patients’ preferences regarding the treatment options. Previous research has shown that patients value effective communication between HCP and patients in addition to technical measures of quality [29]. Although reliable measures for assessing patients’ experiences and perspective do exist, they are not widely incorporated into quality assessment [30]. Knowing patients’ experiences with their HCP is important, as there is evidence showing the link between positive attitudes of patients towards their HCP and improved patient outcomes [31]. To facilitate patients’ involvement in the treatment process, it is important to systematically register patient-related information, such as preferences for and experiences with (drug) treatment, in the medical records. Having such information may not only contribute to improved communication between HCPs and patients but will also provide the source for obtaining the type of quality information that patients value most.

The stakeholders’representatives agreed that PQI should be positively formulated to create an encouraging environment, which is considered very important for participation of HCP in quality improvement programs in general. For the preferred aggregation level, we have found a discrepancy between HCPs and external stakeholders. The HCPs were reluctant to share the prescribing quality data on individual PQI level because of mistrust to the external evaluators. This is in line with other studies showing the unwillingness of physicians to share the quality data with the “general public” [32]. The results of our study suggest that allowing legitimate deviations from the recommended treatment might help to minimize this tendency.

### Potential barriers for use of PQI

We found that the lack of information on reasons why the HCPs do not comply with the drug treatment recommendations is a major barrier for effective use of PQI for all stakeholders. This finding echoes the results from other studies showing that adjustment to patients’ case-mix is a concern for physicians when publishing quality information [33,34]. Such concerns from the HCPs’ side are not unsubstantiated, as it has been shown that for a prominent proportion of patients in clinical practice there are legitimate reasons for not prescribing the recommended treatment [35,36].

According to the Donabedian’s Triad Model of healthcare quality assessment [37], prescribing indicators are typical process indicators as they refer to the treatment of patients. In general, process indicators are considered to be less affected by clinical characteristics of patients compared to the outcome indicators [38]. That is particularly true for process indicators that show percentages of patients in whom certain laboratory measurements have been conducted, or who have received a foot or eye exam. With regard to sensitivity to patient case-mix, however, PQI may behave more like outcome indicators. Presence of comorbidities, patients’ age, co-prescribed medications, contraindications, and possible side effects can all be relevant for the prescribing process, and subsequently the scores of PQI. Therefore, use of PQI requires the same caution with regard to patient characteristics as outcome indicators.

### Limitations

There are some limitations to this study. We included the most relevant organizations in the Netherlands that participate in health care quality measurement and improvement, and within these organizations we recruited the employees whose tasks were most closely related to quality-of-healthcare assessment or improvement. However, the opinions of stakeholders elicited in this study may not necessarily be representative for opinions of similar stakeholders in other countries. In addition, we did not involve patients with diagnosis of T2DM, as the topics covered during the interviews required professional knowledge related to quality of prescribed medication and quality of care measurement using quality indicators.

### Suggestions for future research

A further larger quantitative study of stakeholders’ preferences regarding prescribing quality indicators would give fuller and more generalisable results. Such a study of stakeholders' preferences, with higher participant numbers, could also explore the factors that could optimize the use of prescribing quality indicators, in particular for external use.

In further studies, the actual patients with diagnosis of type 2 diabetes could be included to better reflect on the need and preferences of such patients regarding prescribing quality issues.

## Conclusions

Prescribing quality indicators, especially those focusing on undertreatment, might be relevant for all stakeholders, and therefore, could constitute a basis for selection of a uniform set of quality indicators of diabetes care. The specific preferences of different stakeholders need to be studied in larger groups of participants to reflect each stakeholders view. This study suggests that limited use of PQI in external quality improvement programs might be explained by the fact that the score of PQI are influenced by the side effects, contraindications and patient preferences for drug choice, and these are not systematically taken into account at the moment. Development of information systems for documenting the reasons for deviations from the recommended treatment and patient preferences could contribute to a more widespread use of PQI for different aims.

Our study provides an important insight and a number of domains that could be used for development of questionnaires and conducting surveys to select the prescribing quality indicator’s that are relevant for each of the stakeholders including the ways to optimize their use in daily practice.

## Competing interests

The authors declare that they have no competing interests.

## Authors’ contributions

LM participated in data collection, conducted the analysis and prepared the manuscript; JM participated in data collection, analysis, and interpretation; PD and FM_HR participated to the conception and design and reviewed the manuscript; JB originated the study and reviewed the manuscript. All authors read and approved the final manuscript.

## Pre-publication history

The pre-publication history for this paper can be accessed here:

http://www.biomedcentral.com/1472-6963/12/191/prepub
